# Novel Guanidine Compound against Multidrug-Resistant Cystic Fibrosis-Associated Bacterial Species

**DOI:** 10.3390/molecules23051158

**Published:** 2018-05-11

**Authors:** Aamer Saeed, Alejandra Bosch, Marisa Bettiol, Diana L. Nossa González, Mauricio Federico Erben, Yanina Lamberti

**Affiliations:** 1 Department of Chemistry, Quaid-I-Azam University, Islamabad 45320, Pakistan; 2 CINDEFI (UNLP, CONICET-CCT La Plata), Departamento de Química, Facultad de Ciencias Exactas, Universidad Nacional de La Plata, La Plata 1900, Argentina; bosch@quimica.unlp.edu.ar; 3 Sala de Microbiología, Hospital de Niños Sor María Ludovica, La Plata 1900, Argentina; microbiologialudovica@gmail.com; 4 CEQUINOR (UNLP, CONICET-CCT La Plata), Departamento de Química, Facultad de Ciencias Exactas, Universidad Nacional de La Plata, La Plata 1900, Argentina; diana.nossa@uptc.edu.co

**Keywords:** antimicrobials, thioureas, guanidines, drug-resistant, cystic fibrosis

## Abstract

Chronic pulmonary infection is a hallmark of lung disease in cystic fibrosis (CF). Infections dominated by non-fermentative Gram-negative bacilli are particularly difficult to treat and highlight an urgent need for the development of new class of agents to combat these infections. In this work, a small library comprising thiourea and guanidine derivatives with low molecular weight was designed; these derivatives were studied as antimicrobial agents against Gram-positive, Gram-negative, and a panel of drug-resistant clinical isolates recovered from patients with CF. One novel compound, a guanidine derivative bearing adamantane-1-carbonyl and 2-bromo-4,6-difluouro-phenyl substituents (**H-BDF**), showed potent bactericidal activity against the strains tested, at levels generally higher than those exhibited by tobramycin, ceftazimide and meropenem. The role that different substituents exert in the antimicrobial activity has been determined, highlighting the importance of the halo-phenyl group in the guanidine moiety. The new compound displays low levels of cytotoxicity against THP-1 and A549 cells with a selective index (SI) > 8 (patent application PCT/IB2017/054870, August 2017). Taken together, our results indicate that **H-BDF** can be considered as a promising antimicrobial agent.

## 1. Introduction

In recent years, increasing infections due to antibiotic-resistant pathogens have made the formerly routine therapy of many infectious diseases challenging, and in many instances, extremely difficult or impossible to be eradicated [1,2,3]. Multidrug resistance is specially associated with respiratory tract infection in cystic fibrosis (CF) [4] where opportunistic pathogens such as *Pseudomonas aeruginosa*, *Staphylococcus aureus*, *Stenotrophomonas maltophilia* and species of the *Burkholderia cepacia* complex (Bcc) infect patient’s lung and airways. Although for some patients the infection may occur only transiently, their acquisition most typically results in a chronic infection with acute debilitating exacerbations, causing a severe decline in respiratory function which contributes to disease progression and premature mortality [5,6]. In addition, they are important nosocomial pathogens affecting both immunocompetent and immunocompromised patients, and are responsible for a considerable proportion of infections in patients in Intensive Care Units (ICUs) worldwide [7]. Despite the emergence and dissemination of resistant bacteria and the need of more effective therapies, the development of new antimicrobial agents against these life-threatening infections is declining [8]. The impermeable nature of Gram-negative bacteria envelope, and the presence of multiple efflux pumps, in combination with other resistance mechanisms, has made the discovery of new effective antibacterial drugs very difficult [9].

Thioureas as well as guanidines represent two important groups of compounds due to their wide range of application as pharmaceutical agents. They possess a broad biological activity range including anti-inflammatory, anticancer, antiviral, antiparasitic, antifungal and antimicrobial properties [10,11]. Such a diverse range of biochemical behavior can be attributed to their flexible structure and the presence of nitrogen atoms in these molecules that make it possible to bear various substituents. For instance, it is well known that the 1-aroyl-3-(substituted-2-benzothiazolyl) thioureas exhibit potent antibacterial activity [12]. In addition, 1-(benzoyl)-3-(substituted) thioureas are antimicrobial agents [13] and the fluorinated analogues exhibit good antifungal activity [14]. Furthermore, due to efficient resonance stabilization of the charged protonated state, the guanidine groups have a relatively high acid dissociation constant which makes them stronger bases better suited for stable electrostatic interaction with the negative charged membranes of bacteria. This property improves the penetration of guanidine-bearing compounds through membranes and thus their biological activity [15,16]. On the other hand, the introduction of fluorine or appropriate fluorinated groups into organic compounds has advanced over recent decades in medicinal chemistry. The incorporation of fluorine atoms may contribute to increase metabolic stability, binding affinity and lipid solubility, thereby enhancing rates of absorption and transport of drugs in vivo [17,18]. Several studies further indicated that the incorporation of fluor and/or different electron withdrawing groups, such as bromo, chloro, acetyl, and nitro groups, on aromatic rings results in an improvement in antibacterial activity [16,19,20,21].

Taking into account the aforesaid biological and synthetic significance of thioureas and guanidines on one hand, and the multifunctional value of the electron withdrawing groups in drug design on the other, the endeavor of the current work was to investigate the activity of newly synthesized halophenyl substituted thioureas and guanidines against drug-resistant clinical isolates recovered from patients with CF.

## 2. Materials and Methods

### 2.1. Reagents and Equipment

1-adamantane carboxylic acid, thionyl chloride, triethylamine, potassium thiocyanate, mercury(II) chloride and substituted anilines were commercial products (Sigma-Aldrich, St. Louis, MO, USA) and were used as received. Analytical grade (Merck, Kenilworth, NJ, USA) acetone and dimethyl formamide, DMF, were dried and freshly distilled prior to use.

Melting points were recorded using a digital Gallenkamp (SANYO, Moriguchi, Japan) model MPD.BM 3.5 apparatus and are uncorrected. ^1^H and ^13^C NMR spectra were determined in CDCl_3_ at 300 MHz and 75.4 MHz, respectively, using a Bruker spectrophotometer (Billerica, Middlesex, MA, USA). FTIR spectra were acquired by a FTS 3000 MX spectrometer. Elemental analyses were conducted using a LECO-183 CHNS analyzer (LECO Corporation, MI, USA). Thin layer chromatography (TLC) was carried out on 0.25 mm silica gel plates (60 F254, Merck, Darmstadt, Germany). Visualization was achieved by ultraviolet light.

### 2.2. Synthesis of Compounds

**Figure 1:**
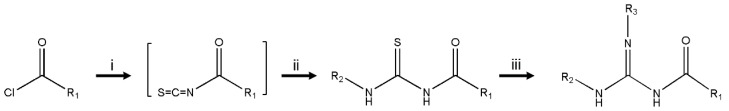
Synthesis of acyl thiourea and guanidine derivatives. Reagents and conditions: (**i**) Acyl chlorides and KSCN in dry acetone, 2 h, reflux. (**ii**) Primary amines in dry acetone. (**iii**) HgCl_2_, substituted aniline and Et_3_N in dry DMF.

Thirteen compounds were synthesized and their structures were confirmed by a combination of elemental analysis, infrared and nuclear magnetic resonance spectroscopy. 1-(Adamantane-1-carbonyl)-3-substituted thiourea compounds were prepared by the addition reaction between adamantyl isothiocyanate with a variety of suitably substituted anilines [22,23,24,25]. The starting material 1-adamantane carbonyl chloride was obtained via the reaction of 1-adamantane carboxylic acid with thionyl chloride. A solution of adamantane-1-carbonyl chloride in dry acetone was treated with an equimolar quantity of potassium thiocyanate in dry acetone to yield the adamantane-1-carbonyl-isothiocyanate as intermediate (Figure 1). A treatment of the latter with an equimolar quantity of cyclohexylamine (for compound **1**, Table 1) and a variety of substituted anilines (compounds **2**–**7**, Table 1) in acetone produced the thiourea derivatives. In a typical procedure, a freshly distilled solution of adamantane-1-carbonyl chloride (10 mmol) in dry acetone (50 mL) was added dropwise to a suspension of potassium thiocyanate (10 mmol) in acetone (30 mL) and the reaction mixture was refluxed for 30 min under nitrogen. After cooling to room temperature, a solution of the substituted aniline (10 mmol) in acetone (10 mL) was added and the resulting mixture refluxed for 2–4 h. The reaction mixture was poured into cold water and the precipitated thioureas were recrystallized from suitable solvents.

Three 1-acyl-3-(2-bromo-4,6-difluoro-phenyl)thioureas (compounds **8**–**10**, Table 1) were synthesized in a similar way by treating the corresponding acyl chloride derivatives (1-naphthoyl chloride, 2,4-dichloro-benzoyl chloride and 4-methyl-benzoyl chloride, respectively) with potassium thiocyanate in dry acetone followed by the addition of 2-bromo-4,6-difluoro-aniline.

**Figure 2:**
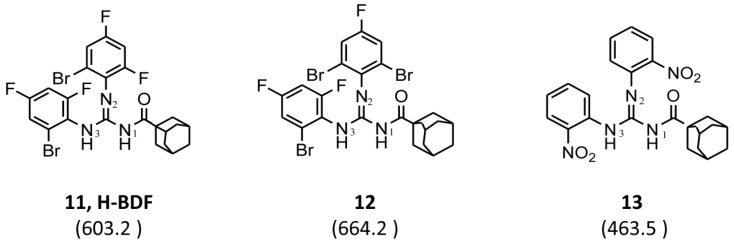
Chemical structure of guanidine derivative compounds **11**–**13**. Molecular weights (g/mol) are shown in parentheses.

For the synthesis of guanidine derivatives (compounds **11**–**13**, Figure 2), the general method proposed by Vencato and coworkers [26] was applied (Figure 1). In a typical procedure triethylamine (2.8 mL, 20 mmol) and selected anilines (10 mmol) were added successively to a stirred solution of the corresponding 1-(adamantane-1-carbonyl)thiourea (10 mmol) in DMF (20 mL) at 10 °C followed by the addition of mercury(II) chloride (2.72 g, 10 mmol). The reaction mixture was stirred at room temperature for 12 h and then filtered to remove the HgS. The filtrate was extracted with EtOAc/H_2_O (1:1) (3 × 5 mL), the organic phase dried over anhydrous Na_2_SO_4_, and concentrated in vacuum to leave an oily residue which recrystallized on standing.

**1-(Adamantane-1-carbonyl)-3-cyclohexylthiourea** (**1**). Yield 68%, semisolid; FT-IR (KBr, ν, cm^−1^): 3336 (NH), 3034 (Ar-CH), 2926 (CH_2_), 2909, 2849 (CH_2_, CH), 1675 (C=O), 1575, 1457, 1370 (C=S). ^1^H NMR (300 MHz, CDCl_3_): δ 13.08 (br s, 1H, NH, D_2_O exchangeable); 6.25 (1H, s, broad, NH); 4.09 (br s, 1H, NH, D_2_O exchangeable); 3.94 (1H, m, CH), 2.1 (br s, 3H, adamantane-CH), 1.95 (s, 6H, adamantane-CH_2_), 1.94–2.02 (2H, dd, CH_2_), 1.60–1.76 (4H, m, CH_2_ × 2), 1.79 (m, 6H, adamantane-CH_2_) 1.18–1.45 (4H, m, CH_2_ × 2); ^13^C NMR (75 MHz, CDCl_3_): 179.1 (C=S); 178.46 (C=O), 54.37 (CH), 41.98, 41.90, 39.2, 38.5, 36.4, 36.0, 33.03 (CH_2_-4), 32.81 (CH_2_-2), 31.6, 28.0, 24.75 (CH_2_-3), 27.7, (adamantane-C) 25.41 (CH_2_-3), 24.75 (CH_2_-3); Anal. Calcd for C_18_H_28_N_2_OS (320.19): C, 67.46; H, 8.81; N, 8.74; S, 10.00%; Found: C, 67.46; H, 8.81; N, 8.74; S, 10.00%.

**1-(Adamantane-1-carbonyl)-3-phenylthiourea** (**2**). Yield 72%, mp 108–110 °C. FT-IR (KBr, ν, cm^−1^): 3336 (NH), 3034 (Ar-CH), 2909, 2849 (CH_2_, CH), 1679 (C=O), 1575, 1457, 1375 (C=S); ^1^H NMR (300 MHz, CDCl_3_): δ 12.71 (br s, 1H, NH, D_2_O exchangeable); 7.63 (br s, 1H, NH, D_2_O exchangeable); 7.23–7.33 (m, 2H, Ar); 7.38–7.43 (m, 2H, Ar), 8.40–8.48 (m, 1H, Ar); 2.08 (s, 3H, adamantane-CH), 1.69 (s, 6H, adamantane-CH_2_), 1.58 (q, 6H, adamantane-CH_2_, *J =* 8.6 Hz); ^13^C NMR (75 MHz, CDCl_3_): 179.6 (C=S); 170.12 (C=O); 143.05 (C-9); 41.51, 39.25, 38.69, 38.49, 36.44, 36.14, 28.05, 27.86, 27.78, (adamantane-C); Anal. Calcd for C_18_H_22_N_2_OS (314.45): C, 68.75; H, 7.05; N, 8.91; S, 10.20%; Found: C, 68.83; H, 7.10; N, 8.98; S, 10.14%.

**1-(Adamantane-1-carbonyl)-3-(4-methyl-3-fluorophenyl)thiourea** (**3**). Yield 69%, mp 174–176 °C. FT-IR (ν, cm^−1^): 3436, 3034, 2909, 1675, 1585, 1457, 1368. ^1^H NMR (300 MHz, CDCl_3_): δ 12.47 (br s, 1H, NH, D_2_O exchangeable); 8.53 (br s, 1H, NH, D_2_O exchangeable); 7.19 (s, 1H, Ar), 7.59 (s, 1H, Ar), 7.81(d, 2H, *J* = 8.6 Hz, Ar), 2.37 (s, 3H, Ar-CH_3_) 2.14 (brs, 3H, adamantane-CH), 1.95 (s, 6H, adamantane-CH_2_), 1.79 (q, 6H, adamantane-CH_2_, *J* = 8.6 Hz); ^13^C NMR (75 MHz, CDCl_3_): 178.9 (C=S), 177.1 (C=O), 161.7 (Ar), 136.7 (Ar), 135.1 (Ar), 136.7 (Ar), 129.7, 141.4, 124.2 (ArCs), 21.2 (Ar-CH_3_) 38.44, 36.14, 27.86, 21.78, (adamantane-C); Anal. Calcd for C_19_H_23_FN_2_OS (346.15): C, 65.87; H, 6.69; N, 8.09; S, 9.25%; Found: C, 65.739; H, 6.72; N, 7.97; S, 9.23%.

**1-(Adamantane-1-carbonyl)-3-(2-nitrophenyl)thiourea** (**4**). Yield 73%, mp 160–162 °C. FT-IR (KBr, ν, cm^−1^): 3336 (NH), 3034 (Ar-CH), 2909, 2849 (CH_2_, CH), 1682 (C=O), 1586, 1543 (NO_2_ asymmetric) 1457, 1368 (C=S), 1340 cm^-1^ (NO_2_ symmetric); ^1^H NMR (300 MHz, CDCl_3_): δ 12.71 (br s, 1H, NH, D_2_O exchangeable); 7.63 (br s, 1H, NH, D_2_O exchangeable); 7.23–7.33 (m, 2H, Ar); 7.38–7.43 (m, 2H, Ar); 2.08 (s, 3H, adamantane-CH), 1.69 (s, 6H, adamantane-CH_2_), 1.58 (q, 6H, adamantane-CH_2_, *J =* 8.6 Hz); ^13^C NMR (75 MHz, CDCl_3_): 179.6 (C=S); 170.12 (C=O); 143.05 (C-9); 41.51, 39.25, 38.69, 38.49, 36.44, 36.14, 28.05, 27.86, 27.78, (adamantane-C); Anal. Calcd for C_18_H_21_N_3_O_3_S (359.44): C, 60.15; H, 5.89; N, 11.69; O, 13.35; S, 8.92%; Found: C, 60.21; H, 5.93; N, 11.71; S, 8.89%.

**1-(Adamantane-1-carbonyl)-3-(4-acetyl-phenyl)thiourea** (**5**). Yield 160–161 °C. FT-IR (KBr, ν, cm^−1^): 3336 (NH), 3034 (Ar-CH), 2909, 2849 (CH_2_, CH), 1679 (C=O), 1575, 1457, 1375 (C=S). ^1^H NMR (300 MHz, CDCl_3_): δ 12.74 (br s, 1H, NH, D_2_O exchangeable), 9.83 (br s, 1H, NH, D_2_O exchangeable), 7.91 (d, 2H, *J=* 8.6 Hz, Ar); 7.73 (d, 2H, *J =* 8.6 Hz, Ar), 2.3 (s, 3H, CH_3_CO), 2.08 (s, 3H, adamantane-CH), 1.69 (s, 6H, adamantane-CH_2_), 1.58 (q, 6H, adamantane-CH_2_, *J =* 8.6 Hz); ^13^C NMR (75 MHz, CDCl_3_): δ 193.6 (CO), 179.6 (C=S), 174.5 (C=O), 143.0, 138.0, 132.6, 127.8, 28.1 (CH_3_), 41.51, 39.25, 38.69, 38.49, 36.44, 36.14, 28.05, 27.86, 27.78, (adamantane-C); Anal. Calcd for C_20_H_24_N_2_O_2_S (356.47): C, 67.39; H, 6.79; N, 7.86; S, 8.99%; Found: C, 67.42; H, 6.83; N, 7.81; S, 8.91%.

**1-(Adamantane-1-carbonyl)-3-(2,3-dichlorophenyl)thiourea** (**6**). Yield 79%, mp 196–198 °C. FT-IR (KBr, ν, cm^−1^): 3336 (NH), 3034 (Ar-CH), 2909, 2849 (CH_2_, CH), 1675 (C=O), 1575, 1457, 1370 (C=S). ^1^H NMR (300 MHz, CDCl_3_): δ 12.74 (br s, 1H, NH, D_2_O exchangeable); 8.70 (br s, 1H, NH, D_2_O exchangeable); 8.03 (d, 1H, *J =* 8.6 Hz Ar), 7.96 (d, 1H, *J =* 8.6 Hz Ar), 7.90 (d, 1H, *J =* 8.6 Hz Ar), 7.83 (d, 1H, *J =* 8.6 Hz Ar), 7.57 (m, 3H, Ar), 2.1 (br s, 3H, adamantane-CH), 2.03 (s, 6H, adamantane-CH_2_), 1.81 (q, 6H, adamantane-CH_2,_ *J =* 8.6 Hz); ^13^C NMR (75 MHz, CDCl_3_): 178.9 (C=S); 134.10 (Ar), 128.6, 126.9 125.3,123.64, 121.67 (ArCs), 41.94, 41.90, 39.2, 38.6, 36.1, 36.0, 31.6, 28.0, 27.8, (adamantane-C); Anal. Calcd for C_18_H_2o_ Cl_2_N_2_OS (383.34): C, 56.40; H, 5.26; N, 7.31; S, 8.36%; Found: C, 56.40; H, 5.26; N, 7.31; S, 8.36%.

**1-(Adamantane-1-carbonyl)-3-(2-bromo-4,6-difluorophenyl)thiourea** (**7**). Yield 70%, mp 194–196 °C. FT-IR (KBr, ν, cm^−1^): 3336 (NH), 3034 (Ar-CH), 2909, 2849 (CH_2_, CH), 1675 (C=O), 1575, 1457, 1370 (C=S). ^1^H NMR (300 MHz, CDCl_3_): δ 11.93 (br s, 1H, NH, D_2_O exchangeable), 9.61 (br s, 1H, NH, D_2_O exchangeable), 7.48–7.44 (m, 1H, Ar), 7.29-7.22 (m, 1H, Ar), 2.08 (t, 10H, adamantane-H, *J* = 6.0 Hz), 1.80 (t, 6H, adamantane-H, *J* = 4.8 Hz); ^13^C NMR (75 MHz, CDCl_3_): 182.3 (C=S), 179.2 (C=O), 163.3, 160.6, 159.8, 157.2, 123.9, 115.8, 104.5, 103.8 (ArCs), 41.9, 37.6, 35.8, (adamantane-C); Anal. Calcd for C_18_H_19_F_2_BrN_2_OS (429.32): C, 50.36; H, 4.46; N, 6.53; S, 7.47; Found: C, 50.24; H, 4.51; N, 6.57; S, 7.36%.

**1-(1-naphtyl)-3-(2-bromo-4,6-difluoro-phenyl)thiourea** (**8**). Yield 81%, mp 174–176 °C. FT-IR (KBr, ν, cm^−1^): 3336 (NH), 3034 (Ar-CH), 1671 (C=O), 1585, 1451, 1372 (C=S). ^1^H NMR (300 MHz, CDCl_3_): δ 11.98 (br s, 1H, NH, D_2_O exchangeable); 11.29 (br s, 1H, NH, D_2_O exchangeable); 8.89–6.71 (m, 9H, Ar); ^13^C NMR (75 MHz, CDCl_3_): 168.9, 164.3, 145.1, 132.0, 134, 120.4, 125.1, 129.6, 116, 103.1 (ArCs); Anal. Calcd for C_18_H_11_ F_2_BrN_2_OS (421.97): C, 51.32; H, 2.63; N, 6.65 S, 7.61%; Found: C, 51.24; H, 2.60; N, S, 6.61, 7.57%.

**1-(2,4-dichloro-phenyl)-3-(2-bromo-4,6-difluoro-phenyl)thiourea** (**9**). Yield 81%, mp 174–176 °C. FT-IR (KBr, ν, cm^−1^): 3336 (NH), 3034 (Ar-CH), 2909, 2849 (CH_2_, CH), 1675 (C=O), 1575, 1457, 1370 (C=S). ^1^H NMR (300 MHz, CDCl_3_): δ 12.07 (br s, 1H, NH, D_2_O exchangeable); 11.35 (br s, 1H, NH, D_2_O exchangeable); 7.61 (s, 1H, Ar), 7.58 (d, 1H, *J =* 8.3 Hz, Ar), 7.58 (d, 1H, *J =* 8.3 Hz, Ar), 7.49 (s, 1H, Ar), 7.19 (s, 1H, Ar); ^13^C NMR (75 MHz, CDCl_3_): 181.9 (C=S); 170.3 (C=O), 168.2, 159.5, 134.10 (Ar), 141.4, 134.1, 130.1, 129.4, 127.3, 128.6, 126.9, 124.2, 119.7, 114.9 (ArCs); Anal. Calcd for C_14_H_17_ Cl_2_ F_2_BrN_2_OS (439.88): C, 38.21; H, 1.60; N, 6.37; S, 7.28%; Found: C, 37.28; H, 1.62; N, 6.33; S, 8.30%.

**1-(4-methylphenyl)-3-(2-bromo-4,6-difluoro-phenyl)thiourea** (**10**). Yield 69%, mp 174–176 °C. FT-IR (KBr, ν, cm^−1^): 3436 (NH), 3034 (Ar-CH), 2909, 1675 (C=O), 1585, 1457, 1368 (C=S). ^1^H NMR (300 MHz, CDCl_3_): δ 12.74 (br s, 1H, NH, D_2_O exchangeable); 11.31 (br s, 1H, NH, D_2_O exchangeable); 7.19 (s, 1H, Ar), 7.59 (s, 1H, Ar), 7.81 (d, 2H, *J =* 8.6 Hz, Ar), 2.51 (s, 3H, Ar-CH_3_); ^13^C NMR (75 MHz, CDCl_3_): 178.9 (C=S), 173.1 (C=O), 134.10 (Ar), 181.7, 141.4, 130.1, 128.6, 126.9, 124.2, 119.7, 114.9 (ArCs), 19.4 (Ar-CH_3_); Anal. Calcd for C_15_H_11_ F_2_BrN_2_OS (385.97): C, 46.77; H, 2.88; N, 7.27; S, 8.32%; Found: C, 46.81; H, 2.92; N, 7.23; S, 8.28%.

**1-(Adamantane-1-carbonyl)-2,3-bis(2-bromo-4,6-difluoro-phenyl)guanidine** (**11**). Yield 70%, mp 148–149 °C. FT-IR (KBr, ν, cm^−1^): 3336, 3413, 3245, 3128, 3043, 3034, 2909, 2849, 1675, 1575, 1457, 1370. ^1^H NMR (300 MHz, CDCl_3_): δ 9.79 (br s, 1H, NH, D_2_O exchangeable); 8.04 (br s, 1H, NH, D_2_O exchangeable); 7.17–7.13 (m, 2H, Ar), 7.06–6.98 (m, 2H, Ar), 2.0 (br s, 3H, adamantane-H), 1.94–1.89 (br m, 3H, adamantane-H), 1.78–1.60 (br m, 10H, adamantane-H); ^13^C NMR (75 MHz, CDCl_3_): 178.2 (C=O), 174.2 (C=N), 154.9, 151.8, 148.6, 131.9, 114.6, 107.8, 103.2 (ArCs), 40.9, 37.9, 35.8, (adamantane-C); Anal. Calcd for C_24_H_21_F_4_Br_2_N_3_O (603.2): C, 47.78; H, 3.51; N, 6.97%; Found: C, 48.1; H, 3.49; N, 7.01%.

**1-(Adamantane-1-carbonyl)-2-(2-bromo-4,6-difluoro-phenyl)-3(2,6-di-bromo-4-fluoro-phenyl)guanidine** (**12**). Yield 70%, mp 144–145 °C. FT-IR (KBr, ν, cm^−1^): 3413, 3245, 3128, 3043, 3034, 2909, 2849, 1675, 1575, 1457, 1370. ^1^H NMR (300 MHz, CDCl_3_): *δ* 11.94 (br s, 1H, NH, D_2_O exchangeable); 9.66 (br s, 1H, NH, D_2_O exchangeable); 7.48 (m, 1H, Ar), 7.23 (m, 2H, Ar), 7.01 (m, 1H, Ar), 1.99–1.84 (m, 10H, adamantane-H), 1.79–1.59 (m, 6H, adamantane-H); ^13^C NMR (75 MHz, CDCl_3_): 179.2 (C=O), 174.2 (C=N), 160.4, 159.8, 157.2, 151.9, 147.2, 140.2, 123.9, 115.8, 114.3, 104.5 (ArCs), 41.9, 37.6, 35.8 (adamantane-C); Anal. Calcd for C_24_H_21_F_3_Br_3_N_3_O (664.2): C, 43.40; H, 3.19; N, 6.33%; Found: C, 43.21.1; H, 3.52; N, 6.97%.

**1-(Adamantane-1-carbonyl)-2,3-bis-(2-nitro-phenyl)guanidine** (**13**). Yield 70%, mp 156 °C. FT-IR (KBr, ν, cm^−1^): 3336, 3413, 3245, 3128, 3043, 3034, 2909, 2849, 1675, 1575, 1457, 1370. ^1^H NMR (300 MHz, CDCl_3_): δ 11.94 (br s, 1H, NH, D_2_O exchangeable); 9.66 (br s, 1H, NH, D_2_O exchangeable); 7.48 (m, 1H, Ar), 7.23 (m, 2H, Ar), 7.01 (m, 1H, Ar), 1.99–1.84 (m, 10H, adamantane-H), 1.79–1.59 (m, 6H, adamantane-H); ^13^C NMR (75 MHz, CDCl_3_): 179.2 (C=O), 174.2 (C=N), 160.4, 159.8, 157.2, 151.9, 147.2, 140.2, 123.9, 115.8, 114.3, 104.5 (ArCs), 41.9, 37.6, 35.8 (adamantane-C); Anal. Calcd for C_24_H_25_N_5_O_5_ (463.5): C, 62.19; H, 5.44; N, 15.11%; Found: C, 61.97.1; H, 5.42; N, 6.93%.

### 2.3. Bacterial Strains

The antibacterial activity of the compounds was tested against the reference strains *Escherichia coli* ATCC25922, *Bordetella bronchiseptica* 9.73H+ [27], *Pseudomonas aeruginosa* ATCC15692, *Burkholderia cenocepacia* J2315, *Pandorea apista* DSM16535, *Staphyloccocus aureus* ATCC6538, *Bacillus cereus* ATCC10876. A total of forty non-fermenting Gram-negative bacilli and two Methicillin-Resistant *Staphylococcus aureus* (MRSA) clinical isolates collected from sputum samples of patients with CF attended at different hospitals and CF Centers in the period 2004 to 2017 were used in this study. They were selected from the collection of microorganisms CAMPA (Colección Argentina de Microorganismos Patógenos y Ambientales) of CINDEFI, at the Faculty of Exact Sciences in La Plata University [28]. All Bcc isolates were identified by PCR-*recA* technology (amplification, PCR-recA RFLP HaeIII, and sequencing). Additionally *hisA*, *gyrB,* or other gene from the current multilocus sequence typing (MLST) scheme were sequenced when the identification remained ambiguous [29,30]. The isolates were maintained both as lyophilized and frozen at −80 °C in Trypticase-soy broth with 10% (*v*/*v*) glycerol until further analysis.

### 2.4. Antimicrobial Activity Assays

The in vitro susceptibility tests (Minimal inhibitory concentration (MIC) and minimal bactericidal concentration (MBC) tests) were determined using the micro-dilution method according to guidelines of the Clinical and Laboratory Standards Institute (CLSI) [31]. Briefly, serial two-fold dilutions of each compound were prepared (final volume of 50 μL) in 96-well polypropylene microtiter plates (Sarstedt, Nümbrecht, Germany) with Mueller Hinton (MH) broth. Each dilution series included control wells without any compound and control wells without bacteria. Then, a total of 50 μL of the adjusted inoculum (approximately 5 × 10^5^ cells/mL) in MH broth was added to each well. The MIC was taken as the lowest concentration of antimicrobial compound resulting in the complete inhibition of visible growth after 18 h of incubation at 37 °C. Minimal bactericidal concentration (MBC) assay was performed following MIC assay. After reporting the MIC assay value, 10 μL aliquots of the medium were taken from wells with no visible bacterial growth. These were plated on LB agar and incubated for 24 h to allow colony growth. The lowest concentration of the compound at which no growth occurred on LB plates was denoted as the MBC. Results are mean values of at least two independent determinations.

### 2.5. Checkerboard Assay

The activity of compound **11** in combination with meropenem, tobramycin and ciprofloxcin was analyzed using the checkerboard broth dilution method [32] to determine the fractional inhibitory concentration indices (FICIs), calculated as: FICI = (MIC**_H-BDF_**^comb^/MIC**_H-BDF_**^alone^) + (MIC_antibiotic_^comb^/MIC_antibiotic_^alone^) (comb, combination). The calculated FICI was interpreted as synergistic (FICI ≤ 0.5), additive (0.5 < FICI < 1), indifferent (1 ≤ FICI < 4.0), or antagonistic (FICI ≥ 4.0), according to the previously published methods [33].

### 2.6. Cytotoxicity Assays

A trypan blue exclusion assay [34] was performed to check the cytotoxicity of compound **11** against THP-1 human monocytic leukemia cells (ATCC, TIB-202, Manassas, VA, USA) and A549 alveolar epithelial cells (ATCC, CCL185, Rockville, MD, USA). Cells were routinely maintained in Complete Medium RPMI-1640 and Dulbecco’s Modified Eagle’s medium (DMEM), respectively, supplemented with 10% heat-inactivated fetal bovine serum (FBS). For the cytotoxicity assay, cells were seeded at a density of 5 × 10^4^ per well in a 96 well plate and were incubated with serial dilutions of compound **11** to a total of 200 μL, at 37 °C in a humidified atmosphere of 5% CO_2_ for 24 h. Two negative controls were included: cells in drug-free culture media and cells treated for 24 h with the maximum concentration of the drug solvent used in the experiment (4% dimethyl sulfoxide). Cells were subsequently stained with 0.2% trypan blue and incubated for 3 min at room temperature. The number of dye-excluding cells was counted by microscopy. A minimum of 200 cells were counted and the percent viability was calculated in comparison to the control. The IC50 value was defined as the highest drug concentration at which 50% of the cells are viable relative to the control. Results are mean values of at least five independent determinations. The selectivity index (SI) was calculated as the ratio of IC50 and the MIC [35].

## 3. Results and Discussion

### 3.1. Chemistry

A series of 11 novel closely related compounds belonging to the thiourea family (compounds **1**–**10**) and a guanidine derivative (**11**) was prepared (Figure 1 and Table 1). Primary amines substituted with different electron withdrawing groups were subjected to the addition reaction with isothiocyanates in order to be transformed into the corresponding thioureas by using the general method originally proposed by Douglas and Dains [22] (Figure 1). The substitution on both nitrogen positions (1 and 3) of the thiourea group was varied in order to better understand the role of different substituents in the biological activity. To rationalize this aspect, a series of closely related 1-(adamantane-1-carbonyl)-3-mono substituted thioureas was firstly prepared by taking into account the well-known capacity of the adamantyl group to enhance antibacterial activity [36,37,38,39,40]. Thus, several thioureas were prepared bearing the adamantyl group in R_1_ (compounds **1**–**7**, Table 1).

Moreover, taking into account the improvement in antibacterial activity exerted by the presence of phenyl groups substituted with electron withdrawing groups [16,19,20,21], a second group of thioureas (compounds **7**–**10**, Table 1) was substituted in R_2_ with the 2-bromo-4,6-difluoro-phenyl group. Finally, the effect of replacing the thiocarbonyl (C=S) with aryl-guanidino functionality (Ar-N=C) was evaluated in compound **7**, in which N-3 of the guanidine was substituted with the 2-bromo-4,6-difluoro-phenyl group. To this end, the procedure proposed by Vencato et al. [26] was applied and the acyl thiourea derivatives were treated with mercury(II) chloride under basic conditions in the presence of 2-bromo-4,6-difluoroaniline to produce the corresponding guanidine derivative (compound **11**, Figure 1 and Table 1) [26,41].

Obtained compounds were purified by flash chromatography. FTIR, ^1^H-NMR and ^13^C-NMR spectra and elemental analysis confirmed the identity of the products (see Materials and Methods). In the ^1^H-NMR of most of the compounds, the characteristic signals of adamantyl moiety: a 6H quartet at δ = 1.75–1.79 ppm (adamantane-CH_2_), a 6H, singlet at 1.95–1.98 (adamantane-CH_2_) and a 3H, singlet around 2.08 ppm (adamantane-CH), besides N-H amide and thioamide singlets at δ = 8.5–8.7 and 12.7–13.0 ppm were clearly observed. In the ^13^C-NMR, characteristic signals for adamantyl moiety at δ = 27.7, 36.1–36.4, 38.6–38.5 and 41.5 ppm, as well those at δ = 170–179 for carbonyl and δ = 178–182 ppm for thiocarbonyl carbons, were observed. The acyl thioureas were also characterized by their IR spectra, with intense absorptions around 3300–3400 (νNH), 1670 (νC=O), 1580 (δNH), and 1380 (νC=S) cm^−1^ [24,42,43].

The guanidine derivative **11** was characterized by two typical NH absorptions at ca. 3400 and 3240 cm^−1^, the C=O stretching at around 1670 cm^−1^ and the absence of thiocarbonyl stretching when the FTIR spectra are compared with the corresponding thiourea reagent. The characteristic C=N stretching modes of the guanidine group are observed as an intense absorption at ca. 1575 cm^−1^. In ^1^H-NMR, two broad NH singlets appeared besides the aromatic protons. The carbonyl carbons are observed at 178–179 ppm in the ^13^C-NMR spectrum, while the (C=N-Ar) appeared upfield at 174 ppm compared to the thiocarbonyl carbon.

### 3.2. Biological Activity

#### 3.2.1. Antimicrobial Evaluation of Newly Synthesized Compounds

All obtained compounds were tested in vitro for their MIC and MBC against two reference Gram-negative non-fermentative bacilli strains, *Pseudomonas aeruginosa* PAO1 and *Burkholderia cenocepacia* J2315. These species play a critical role in morbidity and mortality associated with CF and they were selected on the basis of their high level of resistance to a variety of antimicrobial substances [44,45,46,47]. The results of antimicrobial activity are summarized in Table 1. The MIC and MBC values of meropenem, tobramycin and ceftazidime, three commonly used antibiotics for the treatment of chronic pulmonary bacterial infections [48], were analyzed in parallel. It is apparent from the results that only the guanidine derivative **11**, namely **H-BDF**, showed a MIC value less than 2 µg/mL, and comparable or superior activity than standard drugs. Interestingly, this compound has the lowest MIC and MBC against *B. cenocepacia* J2315, a strain particularly resistant to meropenem [44].

A first look into structural activity relationship (SAR) indicates that, independent of the halogens introduced in the phenyl group, thiourea derivatives have poor or no antimicrobial activity. However, the replacement of thiourea in compound **7** for the guanidine group (compound **11**) greatly improves antimicrobial activity. We next evaluated the impact of introducing changes in the phenyl ring of compound **11** in the biological activity. To this end, the guanidine derivatives **12** and **13** (Figure 2) were synthesized and characterized. Compound **13** was designed to evaluate the effect of changing the substitution of the halophenyl groups by the incorporation of another electron withdrawing group (nitro) in N-2 and N-3, whereas compound **12** evaluates the effect of introducing a small change in N-3 by the substitution of bromine by fluorine in position 6.

The antimicrobial activity of the new compounds was tested against *P. aeruginosa* PAO1 and *B. cenocepacia* J2315 as well as other Gram-negative and Gram-positive reference strains. As shown in Table 2, when the phenyl group substituent of compound **11** was altered by the introduction of a nitro group at the meta position (compound **13**), the guanidine derivate completely lost its inhibition potency, suggesting that not only the guanidine group but also the identity and/or position of the phenyl substitutions are decisive for the antibacterial activity. Moreover, whereas compound **11** exhibited very good inhibitory and bactericidal activity against all tested strains, compound **12**, in which the 2-bromo-4,6-difluoro-phenyl group in N-2 was substituted by 2,6-dibromo-4-fluoro-phenyl ring, showed only moderate microbicidal activity, suggesting that the presence of fluorine atom in position 6 of the phenyl group in N-2 is critical to ensure high inhibition and bactericidal potency.

#### 3.2.2. Cytotoxic Evaluation of **H-BDF**

As limited human cellular toxicity is an important feature for an antibiotic compound, the toxicity of **H-BDF** was evaluated using the human monocytic leukemia cell line THP-1 and the human lung epithelial cell line A549, commonly employed in toxicity evaluation of new compounds for pulmonary application [49,50]. The IC50 for compound **11** was 38.4 ± 5.4 µg/mL for A549 and 15.5 ± 3.1 µg/mL for THP-1 cells. On the basis of the MIC and IC50 values, the selectivity indices were calculated for standard strains (Table 3). It is generally considered that the ratio for a good therapeutic index for a drug should be >10, which is a cut-off point ensuring that overdose does not put the life of the patient in danger [35]. Good SI values were obtained with compound **11** suggesting that **H-BDF** can be considered as a promising antibacterial agent.

#### 3.2.3. Synergistic Effects between **H-BDF** and Conventional Antibiotics

Developments of alternate antibacterial strategies to potentiate the antimicrobial activity of conventional antibiotics have become increasingly important due to the emerging threat of multi-drug resistant infection [51]. As many clinical isolates exhibit resistance to meropenem, ciprofloxacin and tobramycin, three of the different classes of antibiotics commonly used to treat CF pulmonary exacerbations [52], we next studied the ability of **H-BDF** to potentiate the antimicrobial activity of these antibiotics toward the multidrug-resistant strain *B. cenocepacia* J2315. To this end, the relationship between **H-BDF** and meropenem, tobramycin, and ciprofloxacin was assessed via a standard checkerboard assay [29]. Treatment with **H-BDF** reduced the minimum inhibitory concentration of ciprofloxacin and meropenem below their clinical sensitivity breakpoints (≤4 µg/mL and ≤1 µg/mL, respectively). Fractional inhibitory concentration calculations revealed that **H-BDF** exhibited a synergistic interaction with meropenem and ciprofloxacin with FICIs values of 0.3 and 0.4, respectively, and an additive interaction with tobramycin with a FICI value of 0.75. This preliminary study suggests that in addition to being used as antimicrobial agent alone, **H-BDF** has the potential to be used in combination with other antibiotics.

#### 3.2.4. Activity of Compound **H-BDF** against Multidrug-Resistant Clinical Isolates Recovered from Respiratory Samples of CF Patients

Respiratory infections with opportunistic pathogens with intrinsic antibiotic resistance to most clinically available antimicrobials are life-threatening in patients with CF [53,54,55]. Although *P. aeruginosa* and *S. aureus* remain the most common pathogens in CF lung infections, other bacteria such as species within the Bcc, *Stenotrophomonas maltophilia,* and *Achromobacter xylosoxidans,* have emerged as significant opportunistic human pathogens in the last decades [56,57,58,59]. To investigate whether the guanidine derivative **H-BDF** would have clinical utility against current multidrug resistant bacteria, we determined the MIC and MBC of compound **11** against thirty eight Bcc clinical isolates, one *Achromobacter xylosoxidans*, one *Stenotrophomonas maltophilia* and two MRSA recovered from sputum samples of CF patients and selected on the basis of their high level of resistance to a variety of antimicrobial substances [55] (Table 4). MIC values of compound **H-BDF** were generally lower than those of meropenem, ceftazimide and tobramycin. In total, 69% of Bcc clinical isolates had **H-BDF** MIC values less than or equal to 4 µg/mL whereas only 41% of isolates were classified as susceptible to meropenem (MIC values ≤4 µg/mL), 49% were classified as susceptible to ceftazimide (MIC values ≤8 µg/mL), and 2.6% of isolates were classified as susceptible to tobramycin (MIC values ≤4 µg/mL). The activity of compound **H-BDF** against *B. cenocepacia* strains was impressive, with 92% susceptible at 4 µg/mL compared with only 31% susceptible to meropenem at 4 µg/mL, and 69% susceptible to ceftazimide at 8 µg/mL (Table 4)*.* Interestingly, some clinical isolates were resistant to more than 16 antibiotics, such as *B. seminalis* CBC040 [55] had **H-BDF** MIC values ≤ 4 µg/mL. Indeed, **H-BDF** was active against two methicillin-resistant *S. aureus* clinical isolates with MIC values varying from 1 to 2 µg/mL. In conclusion, compound **H-BDF** was active in vitro against a significant number of multi-resistant clinical isolates recovered from CF patients.

## 4. Conclusions

We have reported the synthesis and preliminary evaluation of the antimicrobial activity of 13 novel thiourea and guanidine derivatives. The results evidenced that **H-BDF**, a guanidine derivative bearing adamantane-1-carbonyl and two 2-bromo-4,6-di-fluoro-phenyl groups, can be considered as a promising antimicrobial agent, since it exhibited higher in vitro antibacterial potency against Gram-positive and Gram-negative reference strains than previously reported guanidine compounds [10,11,15]. Moreover, the novel compound was active in vitro against a panel of multidrug-resistant clinical isolates recovered from sputum samples of patients with CF. Preliminary studies further suggest that **H-BDF** was able to significantly potentiate antibacterial synergy with meropenem and ciprofloxacin. From the structure activity relationship, it can be concluded that the antimicrobial activity depends mainly on the presence of a guanidine group. It has been proposed that most of the biological properties of guanidine derivatives are related to their strong basicity due to efficient resonance stabilization of the charged protonated state. The pKa of **H-BDF** was not determined; however, it is expected that under physiological conditions, the guanidine group exists mainly in its protonated form [60]. We can hypothesize that under this state, the guanidine moiety may alter bacterial outer membrane permeability by binding to a negatively charged site in the lipopolysaccharide layer, causing cell death. This mechanism of action have been proposed for several guanidine derivatives with antibacterial activity [61]. Alternatively, the protonated forms may interact with the active site of proteins and enzymes altering its function [11]. By analyzing the role that different substituents exert in the antimicrobial activity, the importance of the halo-phenyl group in the guanidine moiety was also demonstrated. The substituted fluorine in position 6 of the phenyl group in N-2 may contribute to increase binding affinity and/or lipid solubility [18]. Also, the electron-withdrawing group may activate the guanidine binding moiety to enhance its interaction with amine groups present in the cell membrane. Future studies will be directed towards elucidating the targets of **H-BDF** and the mechanisms of action. 

Importantly, this compound displays low levels of cytotoxicity against THP-1 and A549 cell lines. Future research will be performed to evaluate its efficacy and safety in animal models of infection in order to validate its development as a novel antimicrobial.

## 5. Patents

“Antimicrobials compounds”. Patent application PCT/IB2017/054870, August 2017.

## Figures and Tables

**Table 1:** Activities of newly obtained compounds and common antibiotics used in clinical treatments against *Pseudomonas aeruginosa* PAO1 and *Burkholderia cenocepacia* J2315.

Entry	R_1_	R_2_	R_3_	Molecular Weight (g/mol)	Chemical Structure	*P. aeruginosa* PAO1		*B. cenocepacia* J2315	
						MIC (µg/mL)	MBC (µg/mL)	MIC (µg/mL)	MBC (µg/mL)
1	C_10_H_15_^a^	C_6_H_11_		320.19	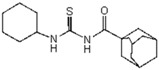	>128	nd	>128	nd
2	C_10_H_15_^a^	C_6_H_5_	-	314.45	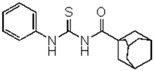	>128	nd	>128	nd
3	C_10_H_15_^a^	3-F-4-CH_3_-C_6_H_3_	-	385.97	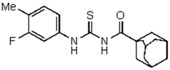	>128	nd	>128	nd
4	C_10_H_15_^a^	2-NO_2_-C_6_H_4_	-	359.44	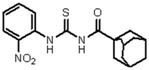	>128	nd	>128	nd
5	C_10_H_15_^a^	4-CH_3_CO-C_6_H_4_	-	356.47	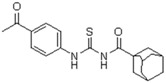	>128	nd	>128	nd
6	C_10_H_15_^a^	2,3-di-Cl-C_6_H_3_	-	383.34	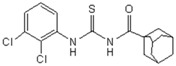	>128	nd	>128	nd
7	C_10_H_15_^a^	2-Br-4,6-di-F-C_6_H_2_	-	428.32	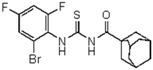	>128	nd	>128	nd
8	C_10_H_7_^b^	2-Br-4,6-di-F-C_6_H_2_	-	421.97	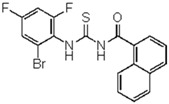	>128	nd	>128	nd
9	2,4-di-Cl-C_6_H_3_	2-Br-4,6-di-F-C_6_H_2_	-	439.88	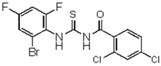	>128	nd	>128	nd
10	4-CH_3_-C_6_H_4_	2-Br-4,6-di-F-C_6_H_2_	-	385.97	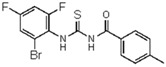	>128	nd	>128	nd
11	C_10_H_15_^a^	2-Br-4,6-di-F-C_6_H_2_	2-Br-4,6-di-F-C_6_H_2_	603.2	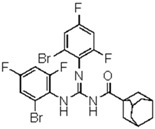	0.5	4	2	8
**Tobramycin**				467.51		2	2	>128	>128
**Meropenem**				383.46		1	4	8	64
**Ceftazimide**				546.57		2	2	16	128

^a^ 1-adamantyl, ^b^-naphthyl

**Table 2:** Anitmicrobial activities of new compounds **11**, **12** and **13** against Gram-negative and Gram-positive bacteria—minimal inhibitory concentrations (MIC, µg/mL) and minimal bactericidal concentration (MBC, µg/mL).

Compound	11 (H-BDF)	12	13	Tobramycin	Meropenem	Ceftazimide
**Organism**	MIC/MBC	MIC/MBC	MIC/MBC	MIC/MBC	MIC/MBC	MIC/MBC
**Gram-negative bacteria**						
*Bordetella bronchiseptica* 9.73H+	0.5/2	16/64	>128/>128	64/64	0.125/0.25	8/64
*Escherichia coli* ATCC25922	1/2	64/64	>128/>128	16/16	0.03125/0.0625	1/1
*Pseudomonas aeruginosa* PAO1	0.5/4	32/>128	>128/>128	2/2	1/4	2/2
*Burkholderia cenocepacia* J2315	2/8	64/128	>128/>128	>128/>128	8/64	16/128
*Pandorea apista* DSM16535	1/2	64/128	>128/>128	32/128	>128/nd	128/nd
**Gram-positive bacteria**						
*Staphyloccocus aureus* ATCC6538	0.25/1	8/64	>128/>128	2/2	<0.125/<0.25	8/8
*Bacillus cereus* ATCC10876	2/2	64/64	>128/>128	8/32	<0.125/<0.25	1/1

nd: no data.

**Table 3:** Selective Indices (SI) of compound **11** against different cell lines.

	Cells	
Organisms	A549	THP-1
**Gram-negative bacteria**		
*Bordetella bronchiseptica* 9.73H+	76.8	30.9
*Escherichia coli* ATCC25922	38.4	15.45
*Pseudomonas aeruginosa* PAO1	76.8	30.9
*Burkholderia cenocepacia* J2315	19.2	7.7
*Pandorea apista* DSM16535	38.4	15.45
**Gram-positive bacteria**		
*Staphyloccocus aureus* ATCC6538	153.6	61.8
*Bacillus cereus* ATCC10876	19.2	7.7

**Table 4:** Microbial susceptibility of multi-resistant isolates recovered from patients with cystic fibrosis.

	H-BDF		Tobramycin		Meropenem		Ceftazidime	
Clinical Isolates ^a^	MIC (µg/mL)	MBC (µg/mL)	MIC (µg/mL)	MBC (µg/mL)	MIC (µg/mL)	MBC (µg/mL)	MIC (µg/mL)	MBC (µg/mL)
***Achromobacter xylosoxidans***								
*A. xylosoxidans* HNA 001	0.125	0.25	R	nd	S	8	S	nd
*Burkholderia cenocepacia*								
*B. cenocepacia* CAMPA 669	0.25	2	S	nd	S	nd	R	nd
*B. cenocepacia* CAMPA 1533	4	16	R	nd	R	64	S	16
*B. cenocepacia* CAMPA 1194	2	4	R	nd	R	nd	R	nd
*B. cenocepacia* CAMPA 544	2	8	R	nd	R	nd	S	8
*B. cenocepacia* CAMPA 1771	8	16	R	nd	I	32	R	nd
*B. cenocepacia* CAMPA 817	2	8	R	nd	R	nd	S	8
*B. cenocepacia* CAMPA 548	2	4	R	nd	R	nd	S	8
*B. cenocepacia* CAMPA 825 (CBC 033) ^b^	4	16	R	nd	I	nd	S	32
*B. cenocepacia* CAMPA538 (CBC 035) ^b^	2	4	R	nd	I	16	S	16
*B. cenocepacia* CAMPA 817	2	8	R	nd	R	nd	S	16
*B.cenocepacia* CAMPA 531	1	4	R	nd	S	nd	S	nd
*B.cenocepacia* CAMPA 993 (CBC 024) ^b^	1	4	R	nd	S	nd	S	nd
*B.cenocepacia* HE001	4	64	R	nd	R	nd	R	nd
***Burkholderia cepacia***								
*B. cepacia* CAMPA 545	4	16	R	nd	R	nd	S	16
*B. cepacia* CAMPA 233 (CBC 012) ^b^	2	4	R	nd	S	8	S	16
*B. cepacia* CAMPA 260	32	nd	R	nd	R	32	R	nd
*B. cepacia* CAMPA 914	32	nd	R	nd	R	32	R	64
*B. cepacia* CAMPA 886	32	nd	R	nd	R	32	R	128
*B. cepacia* CAMPA 998	32	nd	R	nd	R	64	S	32
*B. cepacia* CAMPA 1039	64	nd	R	nd	R	32	R	nd
*B. cepacia* CAMPA 853 (CBC 001) ^b^	32	nd	R	nd	I	64	I	64
*B. cepacia* CAMPA 860 (CBC 007) ^b^	64	nd	R	nd	I	64	R	64
*B. cepacia* CAMPA 660	4	8	R	nd	S	4	R	nd
*B. cepacia* CAMPA 721 (CBC 011) ^b^	2	32	R	nd	S	64	R	nd
***Burkholderia contaminans***								
*B. contaminans* HNBC001	0.25	1	R	nd	R	nd	S	nd
***Burkholderia multivorans***								
*B. multivorans* CAMPA 661(CBC 015) ^b^	2	4	R	nd	S	4	S	8
*B. multivorans* CAMPA 1530	2	8	R	nd	R	nd	S	4
*B. multivorans* CAMPA 647 (CBC 017) ^b^	4	4	R	nd	S	4	S	8
*B. multivorans* CAMPA 653 (CBC 018) ^b^	2	8	R	nd	S	4	S	8
*B. multivorans* CAMPA 623(CBC 019) ^b^	2	8	R	nd	S	8	R	nd
*B. multivorans* CAMPA 832 (CBC 020) ^b^	4	16	R	nd	S	32	R	nd
*B. multivorans* CAMPA 987 (CBC 021) ^b^	2	4	R	nd	S	8	R	nd
*B. multivorans* CAMPA 997 (CBC 022) ^b^	4	8	R	nd	S	8	R	nd
***Burkholderia seminalis***								
*B. seminalis* CAMPA 231	32	nd	R	nd	I	nd	R	32
*B. seminalis* CAMPA 261 (CBC 039) ^b^	32	nd	R	nd	S	16	S	16
*B. seminalis* CAMPA 475 (CBC 040) ^b^	4	8	R	nd	I	nd	R	nd
*B. seminalis* CAMPA 227	1	8	R	nd	R	nd	R	nd
***Burkholderia vietnamiensis***								
*B. vietnamiensis* CAMPA 992 (CBC 038) ^b^	32	nd	R	nd	S	8	S	16
***Staphylococcus aureus***								
*S. aureus* CAMPA 1909	2	16	128	nd	>128	nd	>128	nd
*S. aureus* CAMPA 1908	1	4	32	>128	>128	nd	>128	nd
***Stenotrophomonas maltophilia***								
*S. maltophilia* CAMPA 1911	2	16	>128	nd	>128	nd	>128	nd

nd = non-determined. R= resistant, I = intermediate, S = sensible (according to the criteria set up by the CLSI). Meropenem (≤4 µg/mL S, 8 µg/mL I, ≥16 µg/mL R). Ceftazidime (≤8 µg/mL S, 16 µg/mL I, ≥32 µg/mL R). Tobramycin (≤4 µg/mL S, 8 µg/mL I, ≥16 µg/mL R). ^a^ Isolates recovered from patients with chronic infections in the period 2004–2017. ^b^ Isolates recovered from patients with cystic fibrosis whose complete antibiotic susceptibilities to 17 antimicrobial agents were previously reported (reference [55]).
